# Controlling properties of human neural progenitor cells using 2D and 3D conductive polymer scaffolds

**DOI:** 10.1038/s41598-019-56021-w

**Published:** 2019-12-20

**Authors:** Shang Song, Danielle Amores, Cheng Chen, Kelly McConnell, Byeongtaek Oh, Ada Poon, Paul M. George

**Affiliations:** 1 Department of Neurology and Neurological Sciences, Stanford University School of Medicine, Stanford, CA USA; 2 Department of Electrical Engineering, Stanford University, Stanford, CA USA; 3 Stanford Stroke Center and Stanford University School of Medicine, Stanford, CA USA

**Keywords:** Neural stem cells, Biomedical engineering, Biomaterials - cells

## Abstract

Human induced pluripotent stem cell-derived neural progenitor cells (hNPCs) are a promising cell source for stem cell transplantation to treat neurological diseases such as stroke and peripheral nerve injuries. However, there have been limited studies investigating how the dimensionality of the physical and electrical microenvironment affects hNPC function. In this study, we report the fabrication of two- and three-dimensional (2D and 3D respectively) constructs composed of a conductive polymer to compare the effect of electrical stimulation of hydrogel-immobilized hNPCs. The physical dimension (2D vs 3D) of stimulating platforms alone changed the hNPCs gene expression related to cell proliferation and metabolic pathways. The addition of electrical stimulation was critical in upregulating gene expression of neurotrophic factors that are important in regulating cell survival, synaptic remodeling, and nerve regeneration. This study demonstrates that the applied electrical field controls hNPC properties depending on the physical nature of stimulating platforms and cellular metabolic states. The ability to control hNPC functions can be beneficial in understanding mechanistic changes related to electrical modulation and devising novel treatment methods for neurological diseases.

## Introduction

Neurological diseases and injuries are devastating, often resulting in high morbidity and long-term disability due to the limited regenerative ability of endogenous cells^1–3^. Stem cell therapy has emerged as a promising treatment approach^4,5^ because of cells’ ability to proliferate and the potential to differentiate into one or more lineages under appropriate conditions^6–8^. Specifically, human induced pluripotent stem cells (hiPSCs) reprogrammed from somatic cells have been successfully differentiated into human neural progenitor cells (hNPCs)^9,10^. Transplantation of hNPCs improves functional neurological recovery through increased angiogenesis, dendritic branching and new axonal projections, production of neurotrophic factors, and modulation of the immune system^11–14^. However, the success of hNPC transplantation is currently limited by short-term survival of cells and their failure to integrate with the host tissue^15–17^. It is crucial to understand how hNPCs are influenced by the microenvironmental cues to sustain their viability and potentially enhance the therapeutic efficacy for nerve repair.

The native stem cell niche presents a complex physiochemical microenvironment involving physical (i.e. mechanical stress), chemical (i.e. growth factors), and electrical signals that can dictate stem cell fate and behaviors. Particularly, electrical stimulation plays an important role in biological processes including wound healing, tissue repair, embryogenesis, and remodeling and growth of organisms^18–22^. In the clinical setting, electrical treatment has been applied to revive damaged tissues in the neuromuscular system to accelerate the healing of injured tissues such as nerve, bone, ligament, and articular cartilage^23–26^. The influence of electrical stimulation on stem cells has been widely reported on a variety of cell types and exposure conditions with regard to cell proliferation, differentiation, migration, and ion channel densities^18,19,27–35^. For instance, human mesenchymal stem cells showed increased proliferation and multi-lineage differentiation potential under long-term exposure to pulsed electromagnetic fields from weeks to a month^27,28,34,35^. Electrical stimulation led to longer neurites and greater branching of neural stem cells cultured under differentiation conditions *in vitro*^18,33^. Previous studies also demonstrated that the electrical signal not only guides the migration of neural stem cells *in vivo*^32^ but also leads to their differentiation *ex vivo*^31^. Many studies focused on how the electrical field involved with cell mobilization and long-term differentiation in a voltage or time-dependent manner^18,36–39^. However, there is limited understanding on the effects of the dimensionality of electrical stimulation on hNPCs.

Conductive polymers have gained significant interests due to their ability to interface with neural tissues^22,40–43^. These conductive biomaterials provide a unique platform to study the stem cells function and behaviors electrically. In this study, we have derived scaffolds made of the conductive polymer, polypyrrole (PPy), to understand the effect of changing the dimensionality of electrical stimulating platforms on hNPCs. Specifically, the 2D and 3D PPy scaffolds were electropolymerized and incorporated with hydrogel-immobilized hNPCs. We investigated the physical effect (2D vs 3D) as well as the effect of electrical stimulation on the gene profile of hNPCs. We showed that 2D and 3D electrically-stimulated hNPCs exhibited significant changes in the expression of neurotrophic factors under a brief electrical stimulation. The change in gene expression could be a result of differences in dimensionality of electrical stimulation and nutrient availability associated with the 2D and 3D scaffolds. Neurotrophic factors are known to be important in regulating cell survival, synaptic remodeling and nerve regeneration^44–48^. Therefore, the understanding of the interplay between biophysical cues and electrical signals on hNPCs holds great potential for developing promising therapeutic strategies for nerve regeneration in the future.

## Results and Discussion

### Differentiation of hiPSCs to hNPCs

As described previously, the inhibition by SMAD inhibitors showed effective induction of hiPSCs into hNPCs in the early neuroepithelial progenitor (NEP) phase^9,10^. Following a 7-day induction procedure, a majority of the cells were organized into “rosettes” where the levels of PAX6, SOX1, SOX2, and Nestin were highly expressed at 97.8 ± 7.5%, 99.8 ± 0.7%, 96.8 ± 1.9%, and 97 ± 2.3%, respectively (Suppl. Fig. 1). PAX6, a neuroectodermal marker^49^, is expressed in the regions of the forebrain that give rise to the cortex and functions in the patterning of the brain^50^. A prior study showed that the overexpression of PAX6 favors neural lineage commitment by differentiation into radial glia and subsequently neurons^51^. SOX1 is also one of the earliest expressed neuroectodermal markers^51–53^, and its level of expression increases when NEPs differentiate towards NPCs^54,55^. It is a marker for proliferating NPCs with roles in enhancing and maintaining neuroectodermal commitment^51,56^. Additionally, SOX2 is highly expressed in proliferating NPCs to maintain neural progenitor identity and is downregulated upon further differentiation to neuronal and glial cells^57,58^. Nestin, an intermediate filament protein, expresses exclusively in uncommitted NPCs both *in vitro* and *in vivo*^59,60^. The downregulation of Nestin results in cell differentiation into neurons or glial cells^61,62^. Our immunofluorescent data showed a uniform expression of these markers, indicating the successful differentiation of hiPSCs into hNPCs (Suppl. Fig. 1).

### 2D and 3D hNPCs scaffolds

PPy is one of the most thoroughly investigated conductive polymers for biomedical applications due to its ease for fabrication, high electrical conductivity, and excellent biocompatibility^22,41,42,63,64^. It is known that electrodeposited polymers that are doped with various agents exhibit altered physical, chemical, and electrical properties^41^. Textured surfaces and porous structures could be produced by different electroplating conditions (e.g. lower temperatures) previously described for planar and tubular surfaces^41,42,63^.

**Figure 1:**
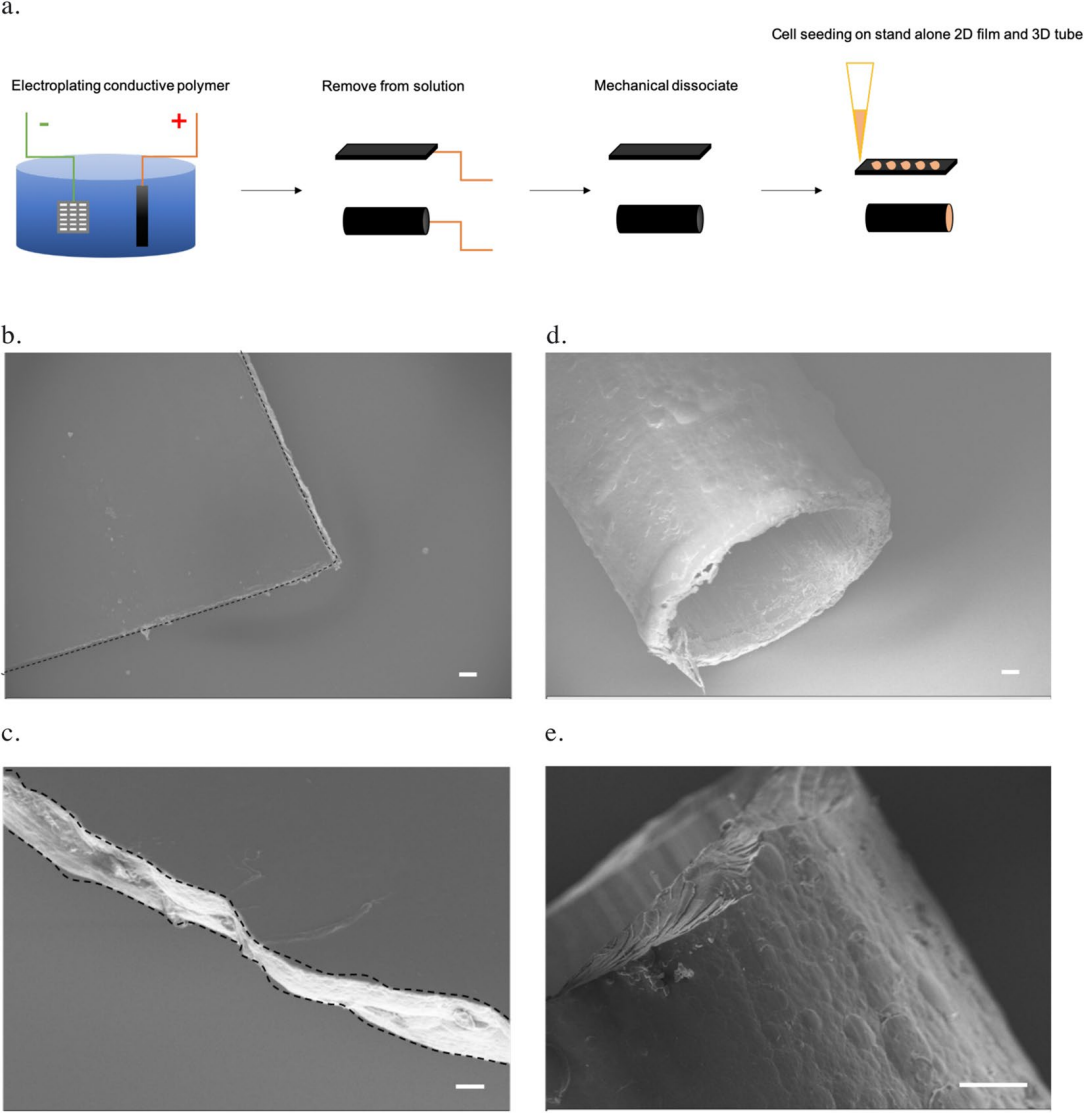
Fabrication and structures of the conductive polypyrrole (PPy) scaffolds. (**a**) Electroplating of the PPy scaffold (black) using platinum mesh (grey) as the reference under applied voltage (green and red as source electrodes) in the NaDBS-doped PPy solution (blue). The PPy scaffolds were mechanically dissociated into 2D films sand 3D tubes, where cells (orange) were deposited on top or inside, respectively. (**b**) Scanning Electron Microscopy (SEM) image showing the top view of the 2D conductive PPy film (outlined in dashes, scale bar: 100 μm). (**c**) The cross-section SEM image of the 2D PPy film (outlined in dashes, scale bar: 20 μm). (**d**) The hollow 3D conductive PPy tube (scale bar: 100μm). (**e**) The side view of sectioned 3D PPy tube (scale bar: 100 μm).

In our study, NaDBS-doped PPy was electroplated at room temperature (Fig. 1a) which resulted in a smooth surface with a mechanically rigid structure for both 2D and 3D conductive PPy scaffolds (Fig. 1b–e). Further inspection showed that the 2D PPy films exhibited a smooth surface morphology (Fig. 1b,c). The inside of the 3D PPy tubes was smooth, whereas the outer surface of 3D PPy tubes was slightly uneven microscopically (Fig. 1d,e). The cross-sectional images showed crystalline structures that were mechanically rigid and insoluble (Fig. 1c,e).

### Modeling the fields produced by 2D and 3D PPy scaffolds

**Figure 2:**
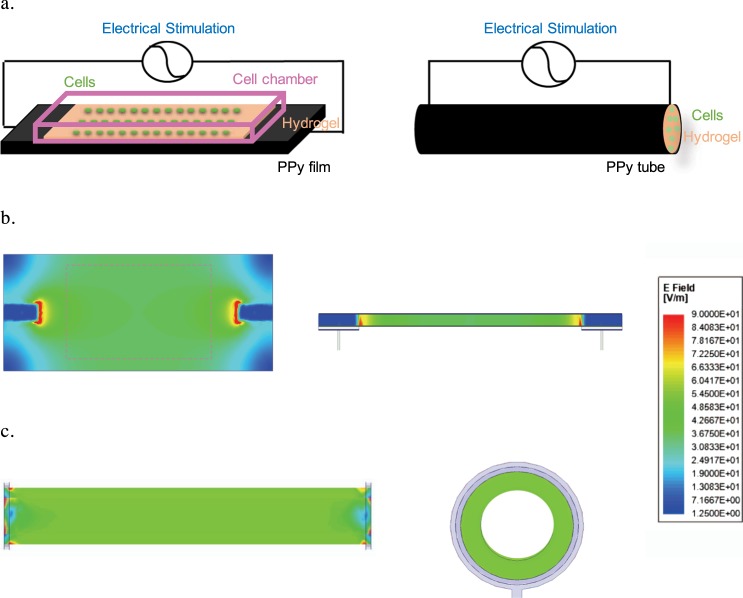
The electrically-stimulated hNPC-seeded polypyrrole (PPy) scaffolds. (**a**) The longitudinal ends of the 2D PPy film (left) or 3D PPy tube (right) were attached with external wires for electrical stimulation. The hydrogel-immobilized cells (green) were seeded directly on top of the 2D PPy film within the cell chamber or encapsulated inside the 3D PPy tube. The Electromagnetic field computation with ANSYS HFSS showed the distribution of the electrical field under applied electrical stimulation in (**b**) the top and side views of 2D PPy film with dashline showing where cells were seeded, and (**c**) the top and cross-section views of 3D PPy tube. The computational modeling indicated that the electrical field was the strongest at the point of contact with the external electrical sources and the electrical fields were uniform across scaffolds with similar field strength (~40 V/m) between the 2D and 3D PPy scaffolds.

The 2D and 3D conductive PPy scaffolds were connected with external wires that allowed for direct electrical stimulation (Fig. 2a). Using an electromagnetic field simulation based on the finite element method for modeling, we observed that sites where the external wires were attached showed a greater field strength. The average field strength for 2D and 3D conditions were 53.6 ± 9.5 V/m and 51.1 ± 2.5 V/m, respectively (Fig. 2b,c). The majority of area in both the 2D and 3D conductive PPy scaffolds exhibited longitudinally and horizontally uniform field strength (~40 V/m) despite the difference in the physical shape of stimulating platform (Fig. 2). Cells were subsequently seeded in those regions with uniform electrical field for both conditions. The Maxwell equation used in this computation employs a homogeneous isotropic medium to model the air and gel, thus not capturing the dimensional variation of the gel’s electrical properties. Moreover, despite the similar electric field strengths, the direction and distribution of the electrical fields are different between the 2D films and 3D tubes. Cells encapsulated in 3D tubes could experience a more unidirectional stimulation signal, whereas cells seeding on 2D films encounter a less uniform stimulation signal due to stronger edge effects. Our stimulating platforms provide a comparable magnitude of electrical stimulation (40 V/m or 0.4 V/cm) to the previous literature. For instance, a carbon nanotube-based scaffold that delivered 0.15 V/cm for 2 ms duration at a frequency of 1 Hz for two weeks re-oriented human mesenchymal stem cells and promoted differentiation towards a cardiac genotype^65^. A graphene-based conductive substrate enhanced the protein synthesis involved in the cell mobility in relation to the cytoskeleton under electrical exposure of 0.045-4.5 V/cm for 32 minutes^38^. Human fibroblasts remained their orientation under one-hour electrical exposure of 2-4 V/cm, but they re-oriented themselves under a stronger electrical field exposure at 7 V/cm^66^. Bone marrow-derived rat mesenchymal stem cells showed no signs of re-alignment with the same electrical setup.

### Viability of hNPCs on conductive scaffolds

**Figure 3:**
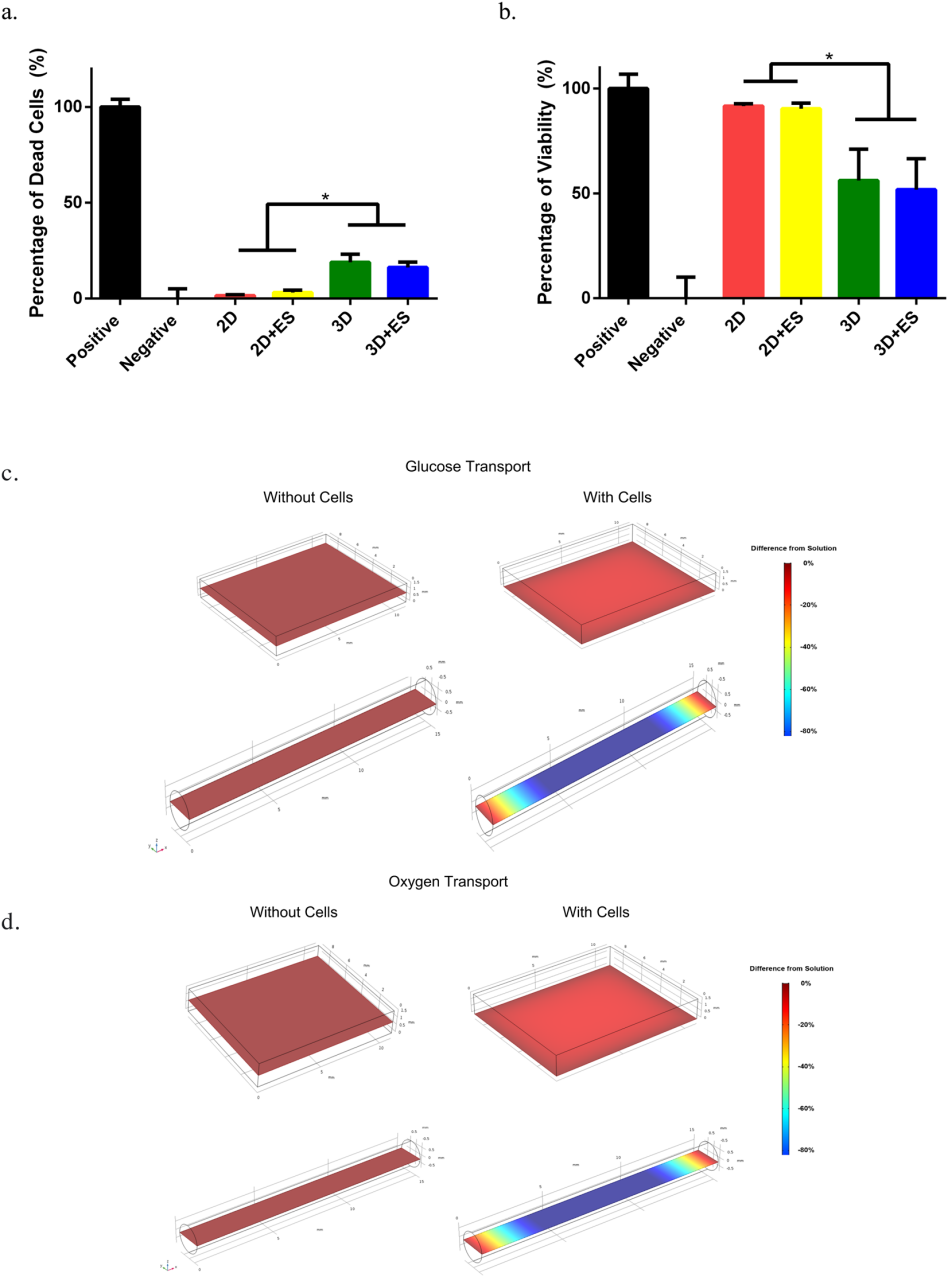
The viability of electrically stimulated hNPCs in the 2D and 3D conductive PPy scaffolds. (**a**) The lactate dehydrogenase (LDH) assay showed no significant difference in the percentage of dead cells caused by electrical stimulation in both unstimulated (2D) and stimulated (2D + ES) 2D PPy films, and unstimulated (3D) and stimulated (3D + ES) 3D PPy tubes, respectively. However, cells encapsulated in the 3D PPy tubes showed significantly higher cell death compared to all 2D conditions. (**b**) The alamar blue assay showed that the viability was similar between the unstimulated (2D) and stimulated 2D PPy films, and the unstimulated (3D) and stimulated (3D) PPy tubes, respectively. In general, all 2D conditions showed better cell viability compared to the 3D conditions. (n = 4, error bars show SE, *p < 0.05) Nutrient molecule transport with COMSOL Multiphysics showed (**c**) glucose and (**d**) oxygen gradient in the 2D PPy film (top) and 3D PPy tube (bottom). With cells (right panels), there were significant changes in nutrient exchange for both scaffolds. The 3D PPy tube showed a significant change in glucose and oxygen concentrations within the tube compared to the 2D PPy film.

After hiPSCs were successfully differentiated into hNPCs, the alginate-immobilized hNPCs were either deposited onto the 2D PPy films or encapsulated into the 3D PPy tubes (Fig. 2a). The total number of hNPCs (~1 million) and the density of cells in the alginate were identical for both platforms. With 1 hr of electrical stimulation of hNPCs on the 2D and 3D PPy scaffolds with identical electrical field strength, we observed that electrical stimulation alone did not change the viability of the hNPCs based on the lactate dehydrogenase (LDH) and alamar blue assays. The LDH assay showed that the percentage of dead cells was 1.6 ± 0.5% and 3.1 ± 1.2% for hNPCs on 2D PPy films, and 19 ± 4.2% and 16 ± 2.8% for 3D PPy tubes under unstimulated and stimulated conditions, respectively (Fig. 3a). The alamar blue assay showed that the cell viability was 92 ± 1.1% and 90 ± 2.6% for unstimulated and stimulated hNPCs on 2D PPy films, and unstimulated and stimulated hNPCs in 3D PPy tubes were 56 ± 15% and 52 ± 15%, respectively (Fig. 3b). Although no significant difference in cell viability resulted from direct electrical stimulation using 2D and 3D scaffolds, we observed that cells on 2D PPy films were more viable compared to the 3D PPy tubes with LDH (unstimulated 2D and 3D cell death: 1.6 ± 0.5% vs 19 ± 4.2%; stimulated 2D and 3D cell death: 3.1 ± 1.2% vs 16 ± 2.8%) and alamar blue assays (unstimulated 2D vs 3D viability: 92 ± 1.1% vs 56 ± 15%; stimulated 2D vs 3D viability: 90 ± 2.6% vs 52 ± 15%). This is likely due to the impermeability of PPy (Fig. 1b–e), which only allowed the axial diffusion of nutrients and waste in the 3D tubes, resulting in a decreased viability compared to those cultured on 2D films. These results were further supported by the modeling of oxygen and glucose transport^67^ for hNPCs on the 2D and 3D PPy scaffolds. Without cells, oxygen and glucose concentrations for 2D films and 3D tubes reached equilibrium with the surrounding culture solution (Fig. 3c,d left panels). However, once cells were placed, the 3D scaffolds showed significant nutrient depletion in the tube lumen, whereas the 2D films exhibited minimal changes in oxygen and glucose gradients (Fig. 3c,d right panels). One limitation associated with modeling cell-incorporated scaffolds is that nutrient consumption was kept at a constant rate, which might not be representative to the real-time cell consumption as the cell survival and subsequent nutrient demand could change over the course of experiments. Another reason for the decreased viability readings from alamar blue assay could be the potential adsorption of reagents in the polymer. Further inspection with live/dead staining showed that hNPCs exhibit the same round cell morphology under unstimulated and stimulated conditions for both 2D and 3D scaffolds (Suppl. Fig. 2).

### Expression changes of electrically stimulated hNPCs in 2D environments

**Figure 4:**
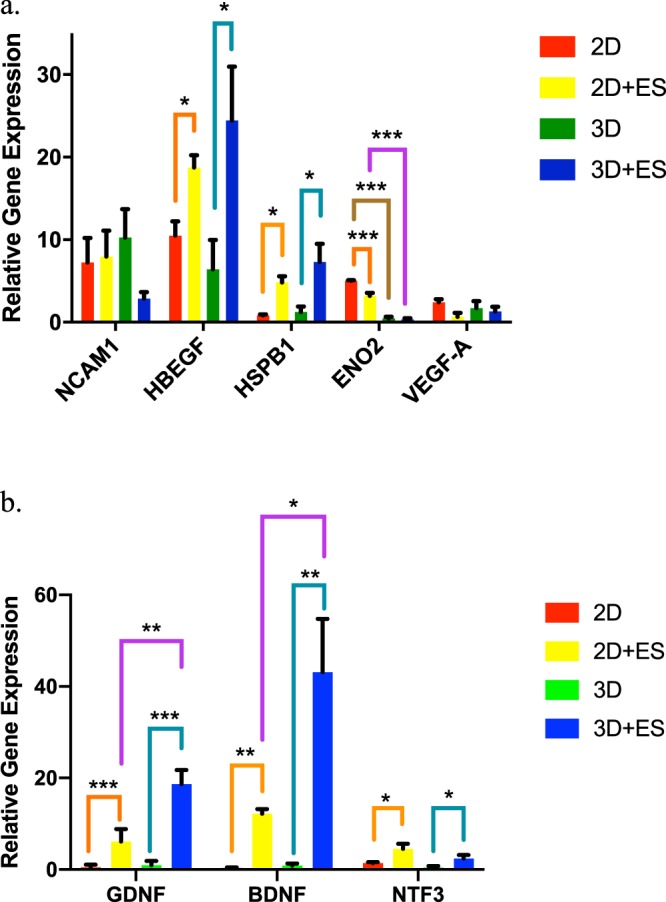
Gene expression changes with electrical stimulation using 2D and 3D conductive PPy scaffolds. The fold change in the relative gene expression of (**a**) NCAM1, HBEGF, HSPB1, ENO2, and VEGF-A and (b) GDNF, BDNF, and NTF3 in unstimulated (2D) and stimulated hNPCs (2D + ES) on 2D PPy films and in unstimulated (3D) and stimulated hNPCs (3D + ES) in 3D PPy tubes. Orange lines indicated comparison between the “2D” and “2D + ES” groups. Teal lines indicated comparison between the “3D” and “3D + ES” groups. Brown lines showed comparison between the “2D” and “3D” groups. Purple lines showed comparison between the “2D + ES” and “3D + ES” groups. (n = 4, error bars show SE, One-way ANOVA followed by multiple comparisons by Tukey, *p ≤ 0.05, **p ≤ 0.01, ***p ≤ *0.001*).

Previous studies demonstrate electrical stimulation causes preferential neurite growth, directional cell migration, and long-term differentiation changes^18,21,33,38,39,68^. Indeed electrical exposure is known to also play a role in induced expression of numerous transcription factors that have great implications in self-renewal and survival, synaptic remodeling, and nerve regeneration^44–47,69^. We hypothesized that the dimensions of stimulating platforms with identical electrical field would impact these changes in hNPCs. Under the electrical stimulation on 2D PPy films, hNPCs showed an upregulation of gene expression in heparin binding EGF like growth factor (HBEGF), heat shock protein family member 1 (HSPB1), glial cell derived neurotrophic factor (GDNF), brain derived neurotrophic factor (BDNF), and neurotrophin 3 (NTF3) and a downregulation of gene expression in enolase 2 (ENO2). The gene expression of HBEGF, HSPB1, GDNF, BDNF, and NTF3 were 1.8-, 5.7-, 12-, 33-, and 3.2-fold higher in the electrically-stimulated hNPCs compared to the unstimulated group under the identical 2D environment, respectively (Fig. 4).

These altered genes play a role in multiple important pathways. Previously, the application of a low electrical field causes upregulation and asymmetric distribution of the epidermal growth factor receptor (EGFR)^70^. Low levels of electrical stimulation caused neural cell precursor proliferation and expression of EGF in the brains of rats^71^. Specifically, HBEGF was shown to stimulate the formation of multipotent glial-derived progenitors in the uninjured Zebrafish retina linking its role in the mediation of the EGFR/MAPK signal transduction pathway^72^. The activation of heat shock proteins (HSP) has been hypothesized as a result of physiological stress induced by electrical stimulation^73^. HSPB1 is a member of the HSP family^74^ with the ability to interact with components of the actin cytoskeleton and protect against cytoskeletal injury during stress^75^. HSPB1 mutations result in the onset of motor neuropathy present with slowly progressive peroneal muscular atrophy, decreased compound muscle action potentials and sensory nerve action potentials based on electrophysiological evidence^76–79^. Neurotrophic factors including GDNF, BDNF, and NTF3 are important regulators in cell fate decisions, axon branching, dendrite pruning, and appropriate patterning of neuronal functions and innervation^44^. They are crucial for the survival, development, and function in the nervous system^44–47,69^. Changes in the expression levels and activities of neurotrophic factors have great implications in many neurodegenerative diseases including Parkinson disease, Alzheimer disease, and Huntington disease^69^.

### Expression changes of electrically stimulated hNPCs in 3D environments

Similarly, the electrically stimulated hNPCs in 3D PPy tubes showed an increase in gene expression of HBEGF, HSPB1, GDNF, BDNF, and NTF3 where they were 3.8-, 5.8-, 19-, 48-, 5.7-fold higher than unstimulated cells, respectively (Fig. 4). Other studies have also demonstrated that electrical stimulation enhanced neurological recovery by modulating the secretion of neurotrophic factors^22,64,80–82^. For example, PPy coated poly(l-lactic acid-co-caprolactone) (PLCL) conduits showed upregulation of genes GDNF, BDNF, and NTF3 on dorsal root ganglia (DRG) cells under electrical stimulation of 100 mV/cm for 4h^82^. Direct stimulation (50–1000 mV/mm) on rat Schwann cells through PPy and chitosan composite also demonstrated an increase in BDNF level *in vitro*^80^. The hollow composite made of PPy and chitosan with electrical stimulation (3 V, 20 Hz, 1 hr) further promoted transected nerve regeneration and functional recovery in rats for 12 weeks^81^. Our findings show that HBEGF, HSPB1, GDNF, BDNF, and NTF3 were upregulated as a direct result of electrical stimulation regardless the shape of the stimulating platform used. Given the importance of these factors for neurotrophic effects, electrical stimulation on hNPCs through our conductive 2D and 3D scaffolds could provide beneficial therapeutics to potentially treat neurological disorders.

Interestingly, electrically-stimulated hNPCs on 2D PPy films showed that the expression of ENO2 was 0.64-fold lower than the unstimulated group, whereas no changes were observed for ENO2 in the hNPCs seeded in 3D PPy tubes under unstimulated and stimulated conditions (Fig. 4a). No changes in gene expression of VEGF-A were observed for all experimental groups (Fig. 4a). ENO2 is a hallmark of neuronal lineage related gene in neural stem cells (NSCs)^83,84^. VEGF-A is important for angiogenesis, cell proliferation, and plasticity^22,85,86^. Our results were different from the previous studies that reported highly expressed ENO2 in neural stem cells (NSCs) after 1 week regardless the use of electrical stimulation (5 mV, 0.5 mA, 25 ms intermittent stimulation) on conducting ropes^83^ and increased VEGF-A expression of hNPCs on polypyrrole surface (2 V, 1 kHz, 1 h)^22^. These discrepancies in findings could be due to differences in the experimental materials (e.g. cell source, conductive substrate, scaffold shape) and methods (e.g. stimulating parameters, culture conditions) used in the literature. Neural cell adhesion molecule (NCAM), expressed on the cell surface of various cell types like neurons and glial cells^87^, was found to express at similar levels in all samples (Fig. 4a), suggesting a potential enhancement in cell-cell adhesion, cell proliferation and directional growth, and synaptic plasticity potential^87–90^.

### Expression changes of hNPCs in 2D and 3D environments

We further investigated the physical effect of 2D and 3D environment on hNPCs alone. In the long-term (~ 2 weeks), hNCPs stemness varies as function of their surrounding hydrogel stiffness and degradability^91^. In our study, the alginate-immobilized hNPCs that were seeded on the 2D surface showed significantly higher expression of ENO2 without electrical stimulation (Fig. 4a). Previous literature reported that ENO2 expression increases as a result of 3D culture^92^ and is a key glycolytic enzyme^93,94^. Our transport modeling indicated that different dimension of cell encapsulation (2D vs 3D) significantly impacts cell survival (Fig. 3c,d). Therefore, the physical microenvironment, in the short term, could directly affect the metabolic pathway of hNPCs, which would be important to consider when designing cell-seeded conductive scaffolds.

### Expression changes of electrically stimulated hNPCs in 2D and 3D environments

More importantly, we examined whether the shape of the stimulating platform (2D vs. 3D) was more effective in promoting certain gene expressions in hNPCs during a short period of electrical stimulation. Shown previously, the long-term electrical modulation can effectively bias the differentiation of stem cells preferentially into a certain lineage (neuronal lineage)^95^. Our findings showed that 3D electrically-stimulated hNPCs were more effective in enhancing neurotrophic factor expression namely GDNF and BDNF (Fig. 4b), whereas 2D electrically-stimulated cells increased gene expression of ENO2 (Fig. 4a). Interestingly, the 3D field alters GDNF and BDNF to a far greater degree than the 2D field. This finding illustrates that even under the identical field strength for a brief period of time, the shape of stimulating platforms has great implications in eliciting the therapeutic potential of hNPCs. Future work can be conducted to study the long-term effects of the dimensionality of electrical stimulation on changes in hNPCs gene expression and their implications in cell proliferation and differentiation.

In this study, we demonstrated the feasibility of fabricating the conductive 2D and 3D PPy scaffolds as platforms to examine the potential dimensional effect of electrical stimulation on hNPCs. Specifically, the 2D and 3D PPy scaffolds with similar material morphologies were used to electrically stimulate hNPCs under identical field strength. Electrical stimulation applied through 2D and 3D conductive scaffolds shared upregulated expression of some common genes that play important roles in signaling self-renewal and survival, synaptic remodeling, and nerve regeneration despite differences in the physical shapes of the stimulating platform. The physical 2D and 3D culturing conditions alone also affected the NPC gene expression related to cell proliferation and metabolic pathways. Importantly, the 3D electrically-stimulated cells were more effective in promoting expression of neurotrophic factors and the 2D stimulated conditions showed increased gene expression that is important for cell-cell adhesion, neuronal differentiation, and metabolic maintenance. In addition to different stimulating platforms, the nutrient availability associated with 2D and 3D scaffolds was also likely to contribute changes in the expression of neurotrophic factors. Overall, our study demonstrated the significance in manipulating the physical culture conditions with brief electrical stimulation that resulted in profound effects reflected by the gene expression profile of hNPCs. Thus, the dimension of stimulating platforms used to apply electrical stimulation proved to be important for modulating expression changes of hNPCs depending on their metabolic states. This discovery can be important in implementing engineering strategies to manipulate the microenvironment to obtain desired therapeutic effects from stem cells.

## Materials and Methods

### Fabrication of conductive scaffolds

For 2D PPy conductive films, the electroplating PPy solution was doped with sodium dodecylbenzenesulfonate (NaDBS) which was then electroplated onto prepared indium tin oxide (ITO) slides at 2 mA/cm2 as previously described^22,41,64^ (Fig. 1a). After removal from the ITO, the electroplated-PPy was washed with DI H_2_O. It was sandwiched between polydimethylsiloxane (PDMS; Sylgard, Dow) slab and a chamber slide to form cell chambers on top of the 2D PPy film (Lab-Tek, Thermo Fisher) (Fig. 2,a). The PDMS slab was used to flatten out the 2D PPy films and prevent potential leakage from subsequent cell culture experiments. Wires were attached to the 2D PPy (length: 3 cm, width: 1.2 cm, thickness: 0.25 mm) outside of the chambers. The individual chamber dimension was 1.1 cm in length and 0.9 cm in width. For the 3D PPy conductive tubes, PPy was electroplated onto a 14G Nickel-Chromium Alloy wire at 2 mA/cm^2^ for 2 hrs (Fig. 1a). The 3D PPy tubes were then gently detached from the plating wire with the following dimensions: 1.63 mm inner diameter, 15 mm length, and 0.4 mm in wall thickness. All scaffolds were electroplated with 0.2 M NaDBS and 0.2 M PPy (Sigma Aldrich) at room temperature. To disassociate scaffolds from underlying substrates, −10 V was applied between the substrate and the reference platinum mesh for 2 min via the function generator (E3641A, Agilent) in PBS. All scaffolds were then mechanically removed from the substrates and sterilized under UV light prior to use^42^. The conductivity was measured to be 57.8 ± 4.2 S/m using the direct current (d.c.) four-point probe method with a Keithley 2400 Source Meter 45 at room temperature. Physical dimensions of scaffolds were measured by a caliper.

### Differentiation of hiPSCs to hNPCs

The differentiation of hiPSCs to hNPCs was generated using defined conditions with modifications to previously reported protocols^9,10^. The NPC differentiation medium was made of 50% DMEM/F12 (Thermo Fisher Scientific), 50% Neurobasal (Thermo Fisher Scientific), 1% N2-Max (R&D Systems), 2% B27 (Thermo Fisher Scientific), 1% non-essential amino acids (NEAA) (Fisher), 1% GlutaMax (Fisher), 0.1 mM Mercaptoethanol (Sigma), and 1% penicillin/streptomycin (P/S) (Fisher) supplemented with SMAD inhibitors (i.e. 1 μM Dorsomorphin and SB431542). The NPC maintenance medium consisted of differentiation medium supplemented with bFGF (20 ng/ml) (Fisher) and EGF (20 ng/ml) (Fisher) without SMAD inhibitors.

On day 0, iPSCs (~80–90% confluency) were washed with PBS without Ca^2+^ and Mg^2+^, followed by addition of NPC differentiation medium for 7 days (4 mL per 6-well) under standard culture conditions (37 °C, 5% CO_2_). Medium was replaced every 24 hr. On day 7, after the induction procedure, hNPCs were washed with PBS, and Accutase (1 ml per well) was added to detach the cells. After 5 min, cells were detached from the plate surface under gentle dislodging. All cells were collected into medium made of DMEM/F12 with the RhoA/ROCK inhibitor and spun down at 1,200 rpm for 5 min at room temperature. The number of cells was determined using a hemocytometer. hNPCs were used between passages 1–3.

### Electrically-stimulation of hNPC-seeded conductive scaffolds

Cell density in alginate was kept consistent for both the 2D and 3D conditions. 25,000 cells/ul hNPCs were encapsulated in 1% alginate solution. The solution was dispensed onto the individual chambers of 2D PPy films or encapsulated into the 3D PPy tubes. The thickness of hydrogel on 2D PPy films was 1.5 mm and the radial diameter in 3D PPy tubes was 1.63 mm as measured by a caliper. The hydrogel-immobilized cell solution was also deposited into 48-well plate as controls. The cross-linked solution containing NPC maintenance medium and 1%CaCl_2_ supplemented by the RhoA/ROCK inhibitor was added to all scaffolds under standard culture conditions. New medium was added to all conditions the next day. The NPC-seeded 2D and 3D conductive scaffolds were electrically stimulated to achieve identical electric field strength (~40 V/m) (Fig. 2). After a 1-hr stimulation, all samples were incubated for 24 hrs before analysis.

### Electromagnetic finite element method (FEM) simulation

Electromagnetic field computation was conducted with physical dimensions and electrical properties described in the 2D and 3D PPy scaffolds^42^ (i.e. electrical resistivity of connection ~0.007 *Ωcm* and experimental conductivity 57.8* S/m*). Electromagnetic simulations were performed on ANSYS HFSS using the finite element method (FEM) solver with the model subdivided into many small subsections in the form of tetrahedra. A solution is found such that the interrelated fields within these tetrahedra satisfy the Maxwell’s Equations across inter-element boundaries. Specifically, the electric field ***E*** is solved using the equation $\nabla \times (\frac{1}{{\mu }_{r}}\nabla \times {\boldsymbol{E}})-{k}_{0}^{\,2}{{\epsilon }}_{r}\,{\boldsymbol{E}}=-j\omega {\mu }_{0}{\vec{{\boldsymbol{J}}}}_{source}$, where ${k}_{0}^{2}=\frac{{\omega }^{2}}{{c}^{2}}$, and ${{\epsilon }}_{r}$, *μ*_*r*_ are the relative permittivity and permeability respectively. This equation makes no approximation from Maxwell’s Equations, thus accurately capturing the electromagnetic field within the model. At each iterative calculation, the fields and associated S-matrix is generated, with the next iteration minimizing the field errors with an adaptive mesh refinement process. A solution is found when Δ*S*_max_ is smaller than the target, which is set to be 0.05% for high precision.

### Nutrient molecule transport finite element method (FEM) simulation

Nutrient molecules transport simulations were performed on COMSOL Multiphysics using the finite element method (FEM) solver, with the model subdivided into many small subsections. A solution is found such that the nutrient concentrations within these subsections satisfy the steady-state reaction-diffusion equations. Specifically, concentration ***C***_***i***_ is solved using the equation **∇** (***D***_***i***_**∇*C***_***i***_) = ***R***_***i***_, where ***D***_*i*_ is the diffusion coefficient for the ***i***^*th*^ nutrient, and ***R***_***i***_ is the nutrient consumption rate by the cell culture. Concentration for oxygen and glucose at the hydrogel boundary were set to be the same as in the cell culture medium provided by the manufacture. Diffusion coefficients for oxygen and glucose are set to be 2.5 × 10^−9^ m^2^/s and 1 × 10^−9^ m^2^/s, respectively^67^. Cell numbers from experiments and cell consumption rate per cell values (oxygen and glucose) from previous literature^67^ were used for modeling 2D PPy film sheet and 3D PPy tube.

### Cell viability

For the lactate dehydrogenase (LDH) (Pierce, Thermo Fisher Scientific) assay, the positive control was a sample medium collected from cells that were lysed with a buffer reagent provided by the manufacturer. The negative control was medium from cells cultured on a tissue culture plate under standard culture conditions. Supernatant from unstimulated and stimulated cells in 2D and 3D PPy scaffolds were also collected. Supernatant from all conditions was then mixed with the reaction mixture and later added with the stop solution. The LDH activity was measured by the Spectra Max M2 plate reader (Molecular Devices) at an absorbance of 490 nm and 680 nm, based on the manufacturer’s protocol. For the alamar blue assay, the positive control was cells cultured on a tissue culture plate under standard culture conditions, whereas the negative control was lysed cells. A 10% alamar blue reagent (DAL1025, Thermo Fisher Scientific) was added to each culture condition including the unstimulated and stimulated cells in the 2D and 3D PPy scaffolds. The activity from the alamar blue assay was quantified with the plate reader by monitoring the absorbance of the reagent at 570 nm while using 600 nm as a reference wavelength based on the manufacturer’s protocol. Live/Dead assay was used to stain cells based on the manufacturer protocol (L3224, Thermo Fisher Scientific).

### Immunofluorescence staining

Cells were fixed with 4% formaldehyde followed by PBS washes, permeabilized with 0.1% Triton X-100 for 10 min, and incubated in blocking solution (PBS, 1% normal goat serum) for 30 min. Samples were incubated with primary antibodies (PAX6: 42-6600, SOX1: AF3369, SOX2: AF2018, Nestin: ABD69MI; Fisher Scientific) at a dilution of 1:100-1:300 for 1 hr and washed twice for 5 min with PBS to remove residues. Another incubation with secondary antibodies (Alexa Fluor 555: A27039, Alexa Fluor 488: A-11008; Thermo Fisher Scientific) at a dilution of 1:1000 for 1 hr was used, followed by PBS washes for 5 min. DAPI (1:1000, D9542, Sigma-Aldrich) was added for nuclear staining. Images were obtained using a Keyence BZ-X710 microscope equipped with full BZ acquisition and analysis software.

### Quantitative gene expression

The quantitative real-time polymerase chain reaction (qRT-PCR) was performed using the RNeasy Mini Kit (Qiagen) based on the manufacturer’s protocol. The iScript cDNA Synthesis Kit (Bio-Rad) was used for cDNA synthesis. The QuantStudio 6 Flex Real-Time PCR System (Thermo Fisher Scientific) was used to perform quantitative real-time PCR. Taq polymerase and Taqman primers (Thermo Fisher Scientific) glyceraldehyde-3-phosphate dehydrogenase (*GAPDH* Hs02786624_g1), neural cell adhesion molecule 1 (*NCAM1* Hs00941830_m1), heparin binding EGF like growth factor (*HBEGF* Hs00181813_m1), heat shock protein family member 1 (*HSPB1* Hs00356629_g1), enolase 2 (*ENO2* Hs00157360_m1), vascular endothelial growth factor A (*VEGF-A* Hs00900055_m1), glial cell derived neurotrophic factor (*GDNF* Hs01931883_s1), brain derived neurotrophic factor (*BDNF* Hs02718934_s1), and neurotrophin 3 (*NTF3* Hs00267375_s1) for formed the PCR reaction mixtures. The Delta-Delta CT method was utilized for analysis with the *GAPDH* housekeeping gene and alginate-immobilized hNPCs on tissue culture wells as references.

### Statistical analysis

Sample pairs were analyzed using the Student’s t-test. Multiple samples were evaluated with one-way or two-way analysis of variance (ANOVA) followed by Tukey and multiple comparisons using GraphPad Prism software (San Diego, CA). A p value of <0.05 was accepted as statistically significant for all analyses.

## Supplementary information

Supplementary information 

## Data Availability

The authors declare that all the data in this manuscript are available.
