# Supplementary material for: Controlling properties of human neural progenitor cells using 2D and 3D conductive polymer scaffolds
Source: Sci Rep. 2019 Dec 20;9:19565. doi: 10.1038/s41598-019-56021-w (PMC6925212; doi:10.1038/s41598-019-56021-w)
Supplement: Supplementary file 1 — Supplementary information [file 41598_2019_56021_MOESM1_ESM.docx]

**Title**: Controlling properties of human neural progenitor cells using 2D and 3D conductive polymer scaffolds

**Authors**: Shang Song^1^, Danielle Amores^1^, Cheng Chen^2^, Kelly McConnell^1^, Byeongtaek Oh^1^, Ada Poon^2^, Paul M. George^1,3*^

1. Department of Neurology and Neurological Sciences, Stanford University School of Medicine, Stanford, CA, USA
2. Department of Electrical Engineering, Stanford University, Stanford, CA, USA
3. Stanford Stroke Center and Stanford University School of Medicine, Stanford, CA, USA

* Corresponding author

Corresponding Author:

Paul M. George, MD, PhD

Assistant Professor

Department Neurology and Neurological Sciences

300 Pasteur Dr., MC5778

Stanford Stroke Center, School of Medicine

Stanford University

Stanford, CA 94305-5778

Tel: +1 (650) 725-0013

Email: pgeorge1@stanford.edu

**Supplementary Information**

a.

**
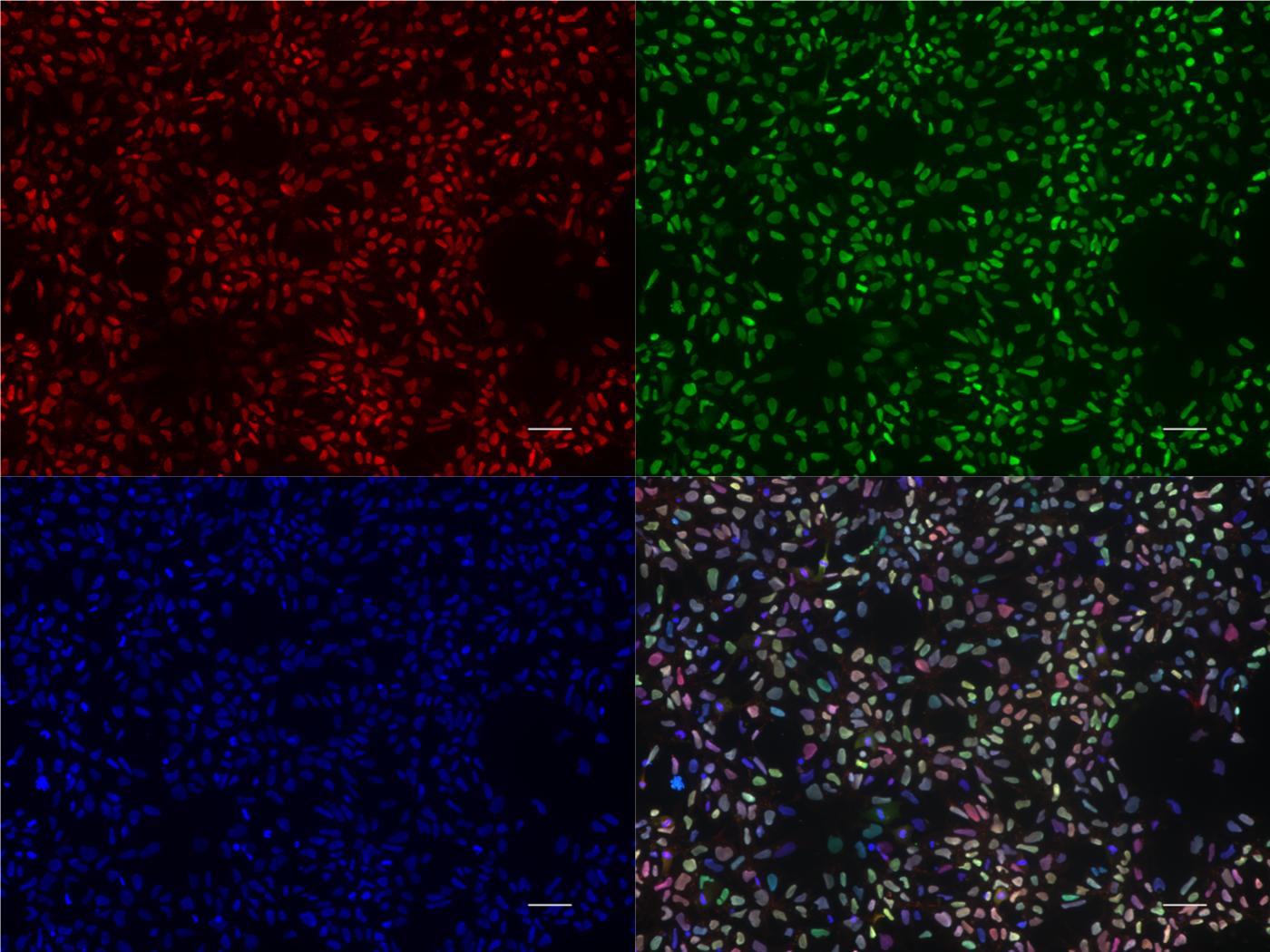
**

b.


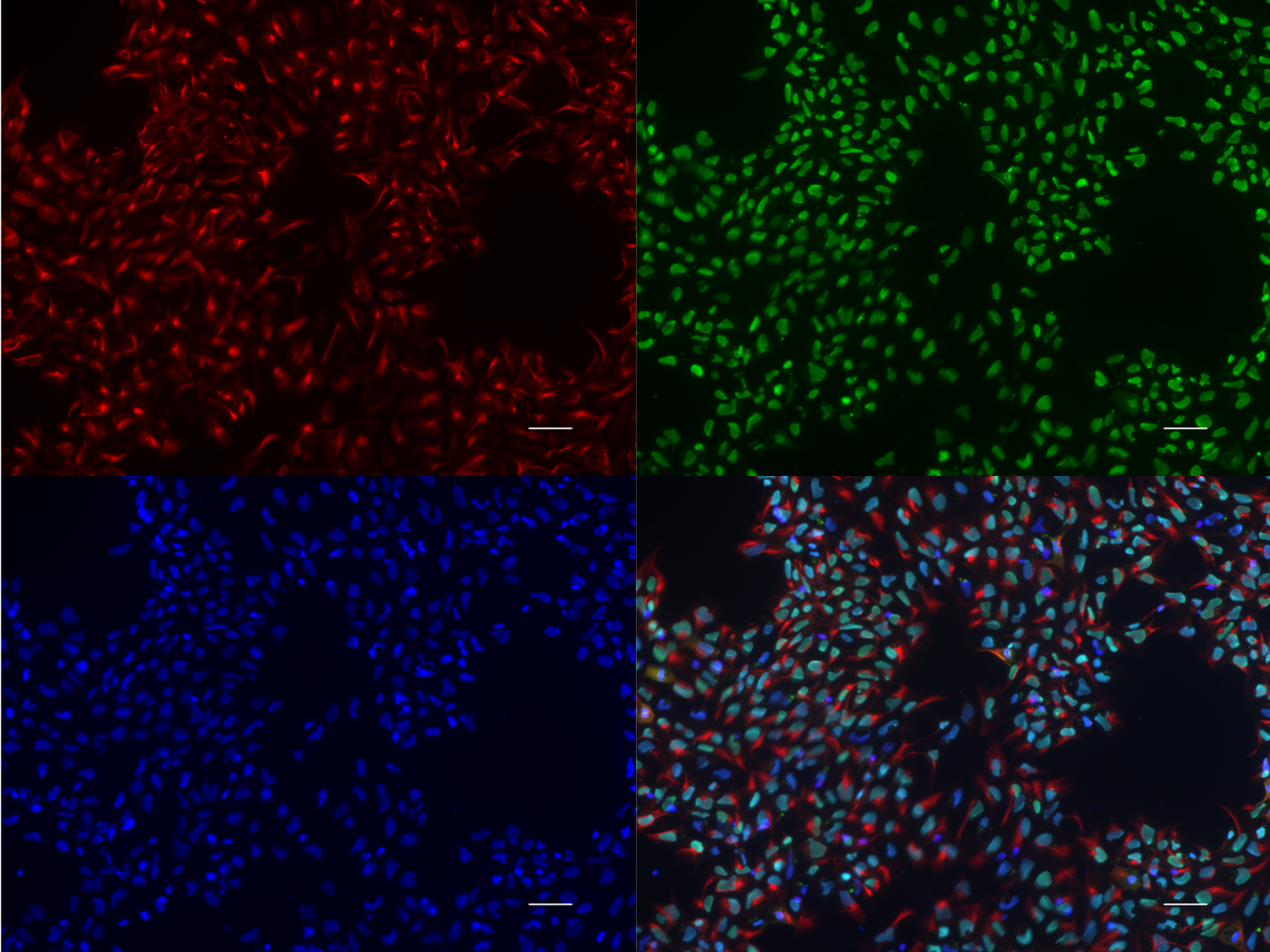


c.

**
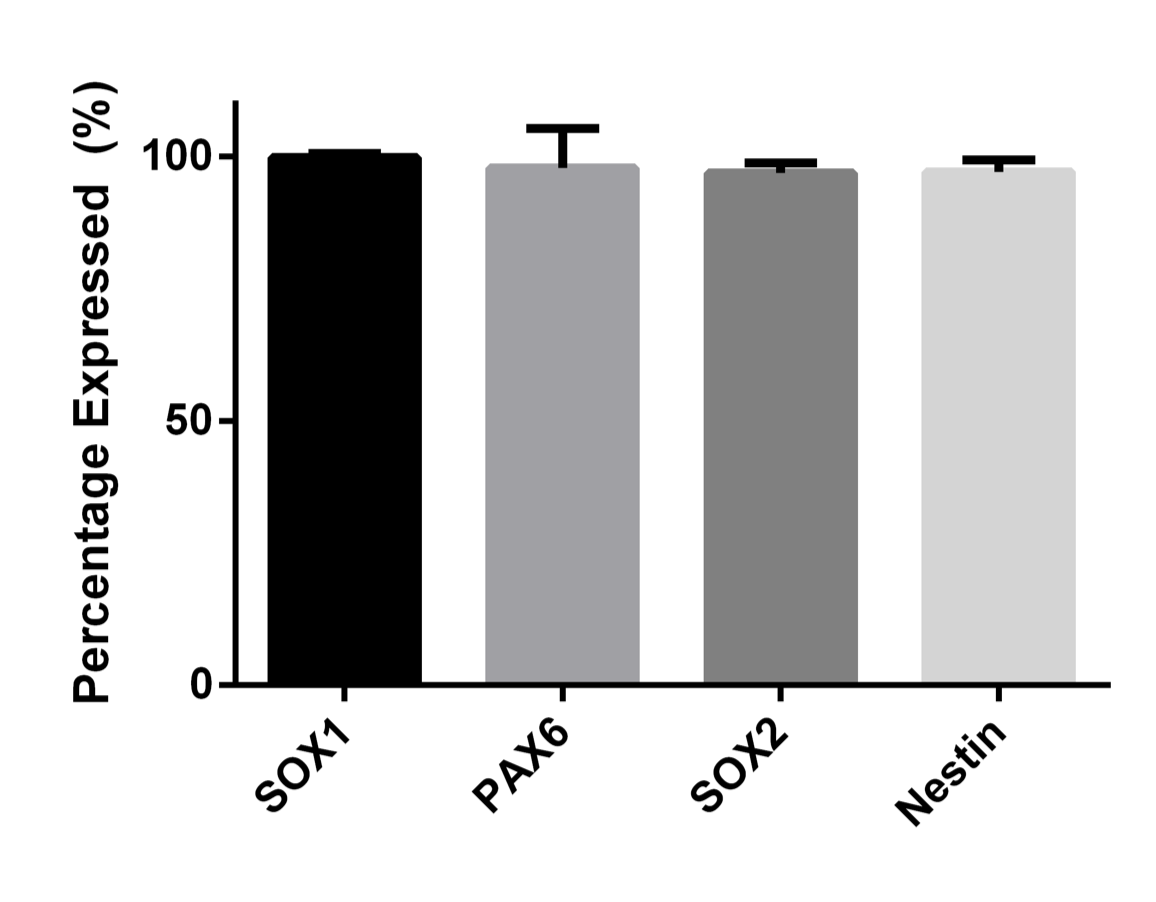
**

Supplementary Figure 1. Immunofluorescence staining of differentiated neural progenitor cells (hNPCs). (a) hNPCs were stained with PAX6 (red), SOX1 (green), and DAPI (blue) (scale bar: 100μm). (b) hNPCs were stained with Nestin (red), SOX2 (green), and DAPI (blue) (scale bar: 100μm). (c) The percentage of the total number of cells that expressed hNPC-related markers indicated that most cells were successfully differentiated into hNPCs with great uniformity (*n=4, error bars show SE*).

Supplementary Figure 2. Live/Dead images of human neural progenitor cells (hNPCs) in the 2D and 3D scaffolds with and without electrical stimulation after 1 day. Live cells were stained in green and dead cells were stained in red. (scale bar: 50μm).
